# Papillomatose confluente et réticulée de Gougerot-Carteaud: à propos d’un cas

**DOI:** 10.11604/pamj.2017.26.207.12166

**Published:** 2017-04-17

**Authors:** Nehal Maja, Ouafa Hocar

**Affiliations:** 1 Faculté de Médecine et de Pharmacie, Université Cadi Ayyad, Service de Dermatologie et Vénéréologie, CHU Mohammed VI, Marrakech, Maroc

**Keywords:** Papillomatose réticulée, papule hyperkératosique, cyclines, Papillomatosis reticularis, hyperkeratotic papule, cyclines

## Image en médecine

La papillomatose confluente réticulée est une dermatose rare du sujet jeune, souvent non ou mal diagnostiquée, asymptomatique, d’étiopathogénie inconnue, caractérisée par la présence de papules hyperkératosiques de couleur brunâtre, de petite taille. Les lésions débutent et confluent dans les régions intermammaire et interscapulaire, puis évoluent de façon centrifuge vers d’autres sites anatomiques où elles prennent un aspect réticulé. L’histologie cutanée montre une hyperkératose, une papillomatose, une acanthose modérée, un discret infiltrat dermique superficiel périvasculaire lymphohistiocytaire, ainsi qu’une discrète hyperpigmentation de la couche basale. Les colorations PAS et Rouge Congo sont systématiques de même que le prélèvement mycologique. Les principaux diagnostics différentiels sont le pityriasis versicolor, l’acanthosis nigricans, la maladie de Darier, l’amylose maculeuse et pigmentée et la dermite séborrhéique. Les traitements proposés sont multiples dont le chef de fil est représenté par les cyclines. Nous rapportons le cas d'une adolescente de 19 ans, sans antécédents particuliers, chez qui l'examen clinique a objectivé des papules kératosiques évoluant depuis 3 ans, de couleur brun jaunâtre, de taille variable, intéressant le cou, le décolleté, les régions intermammaire, l’épigastre ainsi que la racine des deux membres supérieurs. A noter que la patiente était traitée a tord comme pytiriasis versicolor récidivant mise sous antifongiques sans amélioration notable. Le Scotch Test est revenu négatif. La biopsie cutanée a confirmé le diagnostic et un traitement à base de cyclines à été instauré à la posologie de 200mg par jour pendant 3 mois. L’évolution a été marquée par une amélioration spectaculaire avec disparition totale de toutes les lésions cutanées.

**Figure 1:**
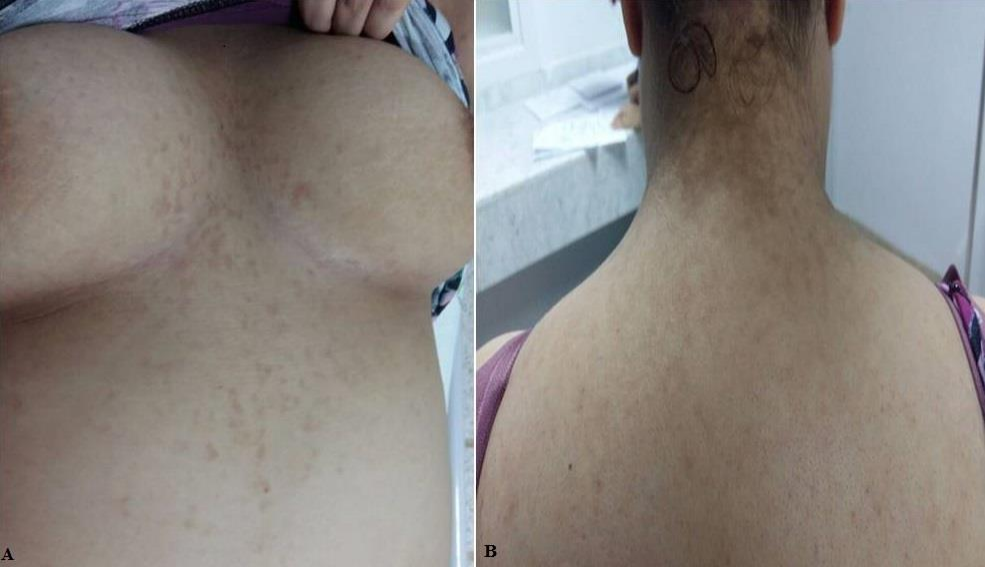
A) papules kératosiques de couleur brun jaunâtres de taille variable de siège inter mammaire, épigastrique et cervical; B) papules kératosiques de couleur brun jaunâtres de taille variable de siège inter mammaire, épigastrique et cervical

